# Platelet Transfusion in Dengue-Associated Thrombocytopenia: A Systematic Review and Meta-Analysis

**DOI:** 10.1590/0037-8682-0181-2025

**Published:** 2026-08-03

**Authors:** Lucca Tamara Alves Carretta, Leonardo Di Cosmo, Lucas Copolillo Faria, Nicole Baptista de Oliveira, Pedro Rodrigues Teixeira, João Vitor Andrade Fernandes, Lara Bourguignon Lopes, Fernando Rocha Oliveira

**Affiliations:** 1 Escola Superior de Ciências da Santa Casa de Misericórdia de Vitória (EMESCAM), Vitoria, ES, Brasil.; 2 Department of Biomedical Sciences, Humanitas University, Pieve Emanuele, Milan, Italy.; 3 Universidade Federal da Paraíba, Centro de Ciências Médicas, João Pessoa, PB, Brasil.

**Keywords:** Dengue, Thrombocytopenia, Platelet transfusion, Hemorrhage, Mortality, Meta-analysis

## Abstract

Thrombocytopenia is a known and potentially severe manifestation of dengue hemorrhagic fever, raising concerns regarding the risk of hemorrhage. To prevent such complications, prophylactic platelet transfusion (PT) has been suggested as a possible intervention. We aimed to evaluate the efficacy and safety of PT in dengue-associated thrombocytopenia. We conducted a systematic literature search for randomized controlled trials and cohorts across PubMed, Cochrane Library, Web of Science, Scopus, and Embase from inception to November 2025. Primary outcomes were time to platelet count ≥ 50 × 10³ platelets/µL and mortality. Secondary outcomes were bleeding events and length of hospital stay. Risk of bias was assessed using the RoB 2 and ROBINS-I tools. Of 1,900 articles screened, 9 studies were included (6 retrospective cohorts, 2 RCTs, and 1 prospective cohort), comprising 3,377 patients. The PT group included 2,383 patients, whereas the standard supportive care group included 994 patients. PT was not associated with significant reductions in time to platelet recovery (mean difference, 0.73 days; 95% CI: -0.01 to 1.47; I² = 95.9%) or length of hospital stay (mean difference, 0.53 days; 95% CI: -0.15 to 1.22; I² = 96.7%) but was associated with significantly higher risks of mortality (risk ratio, 3.61; 95% CI: 1.12-11.66) and bleeding events (risk ratio, 1. 37; 95% CI: 1.03-1.82). Prophylactic platelet transfusion demonstrated no measurable benefit and was associated with increased mortality and bleeding risk, arguing against its routine use in dengue-associated thrombocytopenia. Evidence was primarily derived from retrospective studies, limiting interpretability and warranting randomized studies.

## INTRODUCTION

Dengue is a viral disease transmitted by the bite of an infected *Aedes* mosquito, most commonly *Aedes aegypti*, representing a severe global health problem. Considered the most prevalent among arboviral diseases, it is particularly common in tropical and subtropical regions, such as Brazil^1^. Dengue is endemic in over 100 countries, and its incidence has increased due to various factors, but mainly due to climate change and deforestation^2^. The dengue virus (DENV) has four distinct serotypes (DENV-1 to DENV-4)^1^ and can manifest from asymptomatic forms to potentially lethal cases. Clinically, it is an acute, systemic, and self-limiting pathology^1^, whose severe form is characterized by increased vascular permeability, coagulopathy, and thrombocytopenia^3^. In cases of severe thrombocytopenia, platelet transfusion (PT) may be considered to prevent hemorrhages^3^ ^,^ ^4^.

Dengue management is predominantly supportive, including fluid replacement and symptomatic treatment, as there is no specific antiviral therapy for DENV^1^. During the critical phase, rigorous monitoring and careful administration of intravenous fluids are essential to prevent complications such as hemorrhage and shock^2^ ^,^ ^5^. However, PT remains a controversial practice in the literature, with no consensus on its use. Furthermore, although thrombocytopenia is a frequent complication, there is no clear correlation between low platelet counts and an increased risk of hemorrhage^6^, making transfusion an intervention with limited evidence.

The absence of well-established guidelines for PT in dengue patients with thrombocytopenia results from few conclusive studies in this area^3^ ^,^ ^4^. Several studies that aimed to evaluate the effectiveness of this procedure faced difficulties, especially concerning the understanding of the relationship between thrombocytopenia in dengue and its clinical outcomes, which is still not completely understood^3^ ^,^ ^6^. Additionally, significant heterogeneity is observed among studies due to variations in the criteria adopted to define platelet count thresholds that would justify transfusion^4^ ^,^ ^5^. Given these gaps in current scientific knowledge, the objective of this systematic review and meta-analysis is to comprehensively and methodologically rigorously evaluate the efficacy and safety of PT in patients diagnosed with dengue associated with thrombocytopenia.

## METHODS

This systematic review and meta-analysis was conducted in accordance with the recommendations outlined in the Cochrane Handbook and reported following the Preferred Reporting Items for Systematic Reviews and Meta-Analyses (PRISMA) guidelines^7^ ^,^ ^8^. The study protocol was prospectively registered in the International Prospective Register of Systematic Reviews (PROSPERO; registration number: CRD420251026703).

## ELIGIBILITY CRITERIA

### Inclusion criteria

Inclusion in this meta-analysis was restricted to studies that fulfilled the following criteria: (1) patients with dengue and thrombocytopenia; (2) comparing PT with a control group (standard care); (3) randomized controlled trials (RCTs) or non-randomized cohort studies; (4) reporting one or more of the outcomes of interest; (5) with five or more patients; and (6) published in English.

### Exclusion criteria

To minimize risk of bias, we excluded studies with fewer than four patients, those lacking the outcomes of interest, as well as case reports, reviews, editorials, letters to the editor, conference abstracts, studies not available in English, and studies that did not provide a direct comparison between groups or lacked outcome-specific data.

### Search strategy and data extraction

A systematic literature search was conducted across PubMed, Cochrane Library, Web of Science, Scopus, and Embase databases from their inception until September 2025. The implemented search strategy was: ("dengue" OR "dengue virus" OR "dengue fever") AND ("platelets" OR "thrombocytopenia" OR "low platelet count") AND ("blood transfusion" OR "platelet transfusion" OR "platelet transfusion threshold" OR "platelet count"). Two reviewers (LCF and NBO) independently screened the titles and abstracts, followed by a full-text assessment to determine eligibility. Any disagreements were addressed through consensus in consultation with a third reviewer (LTAC). 

### Data extraction and endpoints

Data extraction was independently performed by two reviewers using a standardized data collection form. The following variables were extracted from each included study: first author and year of publication; study design; study period; study center and country; platelet count threshold for study inclusion (platelets/µL); total number of patients in the PT and control groups; number of patients diagnosed with dengue fever (DF), dengue hemorrhagic fever (DHF), or dengue shock syndrome (DSS); population characteristics, such as pediatric or adult populations, age and sex; and baseline platelet count (×10^3^ platelets/µL). Continuous variables were extracted as mean ± standard deviation, median (range), or median with interquartile range (IQR), as reported. 

### Endpoints

The primary outcomes of interest were time to platelet count ≥ 50 × 10^3^ platelets/µL (days) and mortality. Secondary outcomes of interest included any bleeding events and length of hospital stay. To explore potential sources of heterogeneity and assess the consistency of effects across clinically relevant strata, outcomes were further analyzed in predefined subgroups according to baseline platelet count (platelet count < 20,000/µL versus all platelet counts) and age group (pediatric versus adult populations).

### Risk of bias assessment

The methodological quality of the included RCTs was assessed using the Cochrane Risk of Bias 2 (RoB 2) tool^9^, which evaluates five domains: randomization process, deviations from intended interventions, missing outcome data, measurement of outcomes, and selection of the reported result. The methodological quality of the observational studies included was thoroughly evaluated using the Risk of Bias in Non-Randomized Studies of Interventions (ROBINS-I) tool^10^. This instrument assesses the risk of bias across seven domains: confounding, participant selection, intervention classification, deviations from intended interventions, missing data, outcome measurement, and selection of the reported results. Two reviewers (LCF and PRT) independently carried out the assessments, and the evaluations were subsequently reviewed and validated by a third author (LTAC).

### Statistical analysis

All analyses were performed in R (version 4.5.1) using the meta package. Random-effects models were used throughout to account for between-study heterogeneity, with the between-study variance estimated using the restricted maximum likelihood method. For continuous outcomes, results were expressed as mean differences (MD) with 95% confidence intervals (CI). For binary outcomes, relative risks (RR) with 95% CI were calculated, and P-values < 0.05 were considered statistically significant^7^. Heterogeneity across studies was examined using Cochran’s Q statistic and the corresponding P-value. The degree of inconsistency was quantified with the I² statistic, with values of 0-40%, 40-60%, and > 60% representing low, moderate, and high heterogeneity, respectively^7^. 

### Sensitivity analysis

A leave-one-out sensitivity analysis was conducted to assess the stability of the pooled estimates. This procedure consisted of iteratively excluding each study and recalculating the overall effect size to determine the influence of individual studies on the meta-analysis results. Changes in the pooled MD and corresponding 95% CI were compared across iterations, and substantial alterations after the removal of a particular study were interpreted as evidence of a potentially influential study or outlier.

## RESULTS

### Selection of articles

**FIGURE 1:**
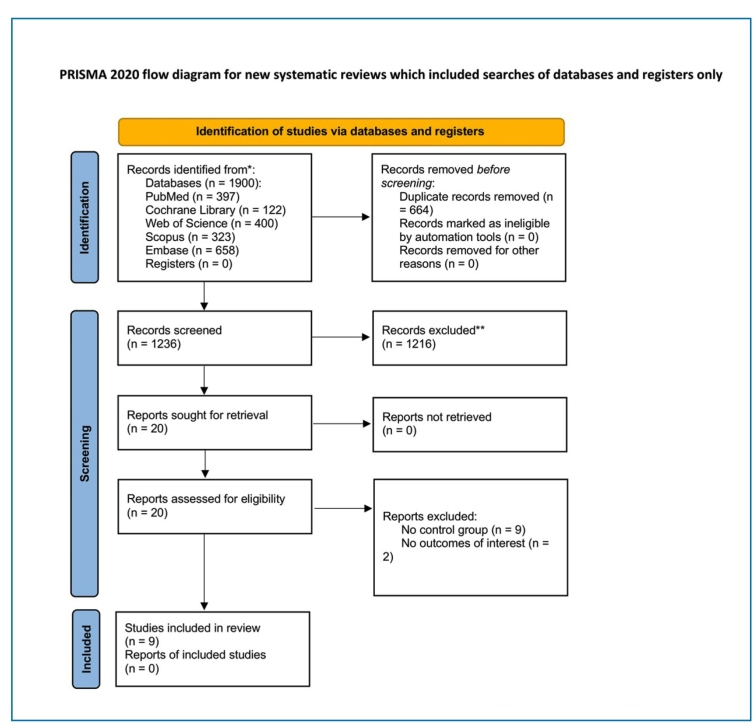
PRISMA flow diagram.

As depicted in the PRISMA flow diagram (Figure 1), this systematic literature search identified a total of 1,900 articles from the five databases: PubMed (n = 397), Cochrane Library (n = 122), Web of Science (n = 400), Scopus (n = 323), and Embase (n = 658). After removing 664 duplicates, 1236 records underwent screening by title and abstract. Of these, twenty-five met the criteria and were sought for retrieval, and all of those were assessed for eligibility. Following full-text assessment, eleven articles were excluded because they lacked a control group (n = 9) or did not report outcomes of interest (n = 2). Ultimately, a total of nine studies were included in the systematic review.

### Baseline characteristics of the included studies

A total of 9 studies were included, with 3,377 patients in total, divided into a control group (n = 994) and a PT group (n = 2,383). Research was conducted between 1994 and 2015 in retrospective cohorts (n = 6), prospective cohorts (n = 1), and RCTs (n = 2). Of these, only two were multicenter, while the other 7 were single-center . The articles originated from diverse countries: Indonesia, Singapore, Malaysia, India, and Pakistan. The study population was divided into adults (n = 5), pediatric patients (n = 2), or both (n = 2). The age of inclusion in the studies ranged from 14 to 21 in general, but one study included all ages. Platelet count for inclusion was also assessed, varying from 20,000 platelets/µL to 50,000 platelets/µL, with three studies lacking platelet count data. 

In most studies, patients were subgrouped according to the dengue manifestation: DF (n = 88), DHF (n = 2,115), DSS (n = 797). The mean age of included patients ranged from 6 to 44.3 years. The male-to-female ratio across the groups varied between 55% and 82.6% in the PT group and between 45.7% and 76.9% in the control group. Mean values for baseline platelet count ranged from 10,000 platelets/µL to 205,000 platelets/µL (Table 1). 

**TABLE 1:** Baseline characteristics of the included studies.

Author, year	Study design	Study period	Center	Country	Pediatric/Adult Populations	Age of inclusion, years	Platelet count for inclusion/mm³	Patients, n PT/control	Patients, n DF/DHF/DSS	Age †, y PT/Control	Male, % PT/Control	Baseline platelet count †, x10³ platelets/mm³ PT/Control
Assir, 2013	RCT	2011 only	SC	Pakistan	Pediatric and Adult	≥ 14	< 30,000	43/44	37/50/0	33 (range 15-65) / 36 (range 16-78)	28 (65.1%) / 29 (65.9%)	10.0 [8.0-15.0] / 10.5 [9-17.7]
Chairulfatah, 2003	RC	1994-1995	MC (4)	Indonesia	Pediatric and Adult	All ages	None	1169/131	0/1198/102	NR	NR	NR
Kabra, 1998	PC	1996 only	SC	India	Pediatric	< 18	< 20,000	18/19	0/37/0	6.65 ± 2.87 / 7.41 ± 2.32	11 (61.1%) / 9 (47.4%)	NR
Lee, 2016	RC	2005-2008	SC	Singapore	Adult	≥ 18	< 20,000	486/302	0/777/11	40 (21-67) / 40 (22-65)	356 (73.3%) / 219 (72.5%)	14.0 (7.0-19.0) / 16.0 (10.0-19.0)
Lum, 2003	RC	1991-2000	SC	Malaysia	Pediatric	< 18	None	60/46	0/0/106	6.0 (0.1-11.0) / 6.0 (0.3-12.0)	33 (55.0%) / 21 (45.7%)	20.5 (5.0-75.7) / 22.0 (8.1-120.6)
Lye, 2009	RC	2004 only	SC	Singapore	Adult	≥ 18	< 20,000	188/68	0/6/256	40 (22-64) / 39 (22-58)	144 (76.6%) / 45 (66.2%)	15.0 (7.0-19.0) / 15.0 (8.0-19.0)
Lye, 2017	RCT	2010-2014	MC (5)	Singapore, Malaysia	Adult	≥ 21	< 20,000	187/182	0/47/322	44.3 (14.1%) / 45.2 (12.4%)	139 (74.3%) / 140 (76.9%)	14.1 ± 5.8 / 13.1 ± 4.6
Prashantha, 2014	RC	2009-2010	SC	India	Adult	≥ 18	< 50,000	23/28	51/0/0	29 (18-38) / 28.5 (18-44)	19 (82.6 %) / 17 (60.7 %)	17.6 (4.0-37.0) / 19.2 (6.0-38.0)
Sethi, 2017	RC	2009-2015	SC	Pakistan	Adult	≥ 21	None	209/430	NR	NR	155 (74.2%) / 274 (63.7%)	NR

**Note. DF:** dengue fever; **DHF:** dengue hemorrhagic fever; **DSS:** dengue shock syndrome; **MC:** multicenter; **NR:** not reported; **PC:** prospective cohort; **PT:** platelet transfusion; **RC:** retrospective cohort; **RCT:** randomized controlled trial; **SC:** single-center; †: mean ± standard deviation, median (range), or median [IQR].

### Outcomes of included studies

**FIGURE 2:**
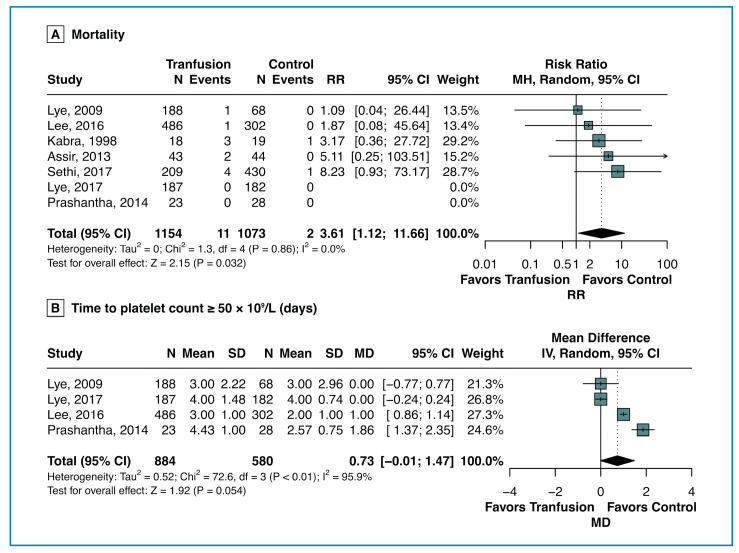
**(A)** Forest plot for mortality and **(B)** Forest plot for time to platelet count ≥ 50 × 10^3^ platelets/µL (days).

*Mortality:* Seven studies (n = 2,227) contributed to this outcome. PT was associated with a higher risk of death compared with the control group (random-effects RR 3.61; 95% CI: 1.12-11.66; P = 0.032; Figure 2A). The findings were similar in the subgroup analysis (Supplementary Figure 1, Supplementary Figure 2). For platelet counts < 20,000/µL, the RR was 1.76, but the association was not statistically significant (95% CI: 0.44-6.98, P = 0.90). When all platelet-count thresholds were considered, the association was not statistically significant (RR = 2.96; 95% CI: 1.19-40.90, P = 0.80). Stratification by population (Supplementary Figure 2) showed only non-significant RRs. The pediatric-only population showed an RR of 3.17 (95% CI: 0.36-27.72); the adult-only population, an RR of 3.52 (95% CI: 0.73-16.94, P = 0.53); and mixed populations an RR of 5.11 (95% CI: 0.25-103.51). Furthermore, all of the mortality outcomes demonstrated no heterogeneity (I^2^ = 0).

*Time to platelet count ≥ 50* × *10* ^*3*^ *platelets/µL (days):* The pooled analysis of 4 studies (Figure 2B) with 1,464 patients, under the random-effects model, demonstrated a non-significant MD of 0.73 (95% CI: -0.01-1.47). This outcome also indicated substantial overall heterogeneity (I² = 95.9%, P < 0.01). 

**FIGURE 3:**
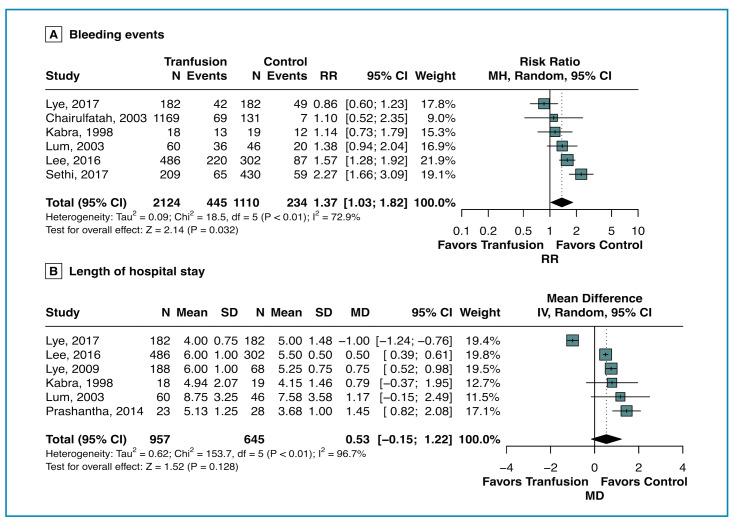
**(A)** Forest plot for bleeding events and **(B)** Forest plot for length of hospital stay.

***Bleeding events:*** Six studies (n = 3,234) contributed to this outcome. PT was associated with a statistically significant higher risk of bleeding events when compared with the control group (random-effects RR, 3.61; 95% CI 1.12-11.66; Figure 3A), with no between-study heterogeneity (I² = 0%; P = 0.032). In the subgroup analysis of platelet count (Supplementary Figure 3), the subgroup with less than 20,000/µL platelets reported a non-significant RR of 1.18 (95% CI: 0.84-1.67, I² = 66.4%, P = 0.03), whereas the subgroup with all platelet counts showed a significant RR of 1.63 (95% CI: 1.06-2.49, I² = 63.6%, P = 0.06). In the population subgroups analysis (Supplementary Figure 4), the pediatric-only population demonstrated an RR of 1.27 (95% CI: 0.95-1.71, I² = 0%, P = 0.52); the adult-only population, an RR of 1.40 (95% CI: 0.54-3.63, I² = 93.8%, P < 0.01); and the mixed adult and pediatric population, an RR of 1.53 (95% CI: 1.26-1.87, I² = 0%, P = 0.38), but only the latter was statistically significant.

***Length of hospital stay:*** Across six studies (n = 1,602), the random-effects meta-analysis did not demonstrate a clear difference in length of stay between both groups (MD 0.53; 95% CI -0.15-1.22; Figure 3B). This outcome demonstrated substantial overall heterogeneity (I² = 96.7%; P = 0.128). When stratified by platelet count (Supplementary Figure 5), the MD among patients with platelet counts < 20,000/µL was 0.21 (95% CI 0.60-1.02; I² = 97.9%; P < 0.01). Regarding all platelet counts, the MD was 1.40 (95% CI 0.83-1.97; I² = 0%; P = 0.71) , without statistical significance. Among the different populations (Supplementary Figure 6), the length of hospital stay was similar. The MD in the pediatric population was 0.96 (95% CI 0.08-1.83; I² = 0%; P = 0.67), and in the adult population, the MD was 0.39 (95% CI −0.41-1.19; I² = 98%; P < 0.01).

### Sensitivity analysis

A sensitivity analysis was conducted, employing LOO and Baujat diagnostics to assess the robustness of the findings, which largely affirmed the main findings but critically highlighted specific studies that exerted a significant influence on pooled estimates and on observed heterogeneity. 

For the time to platelet count ≥ 50 × 10^3^/µL, which exhibited substantial heterogeneity (I² = 95.9%), the LOO (Supplementary Figure 7) and Baujat (Supplementary Figure 8) analyses revealed that the high degree of heterogeneity remained consistently high despite the removal of various studies, suggesting that no single study was solely responsible for the observed inconsistency. For mortality (I² = 0%) and bleeding events (I² = 0%), no between-study heterogeneity was observed. The LOO (Supplementary Figure 9 and Supplementary Figure 10) and Baujat (Supplementary Figure 11 and Supplementary Figure 12) analyses confirmed the robustness of these outcomes. Regarding the length of hospital stay, which also demonstrated substantial overall heterogeneity (I² = 96.7%), the LOO (Supplementary Figure 13) and Baujat (Supplementary Figure 14) diagnostics identified that the Lee et al.^4^ study was a major contributor to the observed variability. Its exclusion in sensitivity analysis led to evident shifts in the pooled MD and considerably reduced heterogeneity, favoring the control group.

These results highlight how the variability caused by diverse patient cohorts, varying definitions of thrombocytopenia thresholds, heterogeneous clinical practices, and reporting across the observational studies were crucial in generating the observed heterogeneity and influencing the sensitivity of the estimates. 

### Risk of bias

Methodological quality was assessed with the ROBINS-I tool, which examines six domains (Supplementary Figure 15). The included studies showed heterogeneous risk profiles across the assessed domains. Most studies were judged to have a low risk of bias regarding participant selection, classification of interventions, deviations from intended interventions, and outcome assessment. However, important limitations were identified in other domains, particularly concerning confounding. Among the seven included studies, three were rated as having a critical risk of bias due to confounding, representing the main source of methodological concern (Chairufatah et al.^11^, Lye et al.^12^ ^,^ and Sethi et al.^13^), and three (Kabra et al.^14^, Lum et al.^15^, and Prashantha et al.^16^) were at moderate risk. Only one, Lee et al.^4^, achieved a low-risk judgment in the first domain. Missing data also showed lingering issues, yielding moderate risk in two of the studies (Lye et al.^12^, Sethi et al.^13^). Consequently, the overall ROBINS-I judgment was moderate in three studies and critical in another three; only Lee et al.^4^ fulfilled criteria for a low overall risk of bias. These findings suggest that, despite the generally acceptable methodological quality of the included studies, certain limitations should be taken into account when interpreting the results of this systematic review and meta-analysis.

The risk of bias of the included RCT was assessed using the RoB 2 tool (Supplementary Figure 16) for two studies (Lye et al.^17^ and Assir et al.^18^). The study by Assir et al. was determined to have some concerns regarding risk of bias, which was attributed to problems in the randomization process and in the selection of the report result. The Lye et al.^17^ study was determined to have low concerns. Quality assessment is illustrated in Supplementary Figure 16.

## DISCUSSION

This systematic review and meta-analysis offers a comprehensive synthesis of comparative evidence regarding platelet transfusion in dengue-associated thrombocytopenia. Across clinically relevant endpoints, platelet transfusion showed no measurable benefit in hematologic recovery or hospitalization duration and was linked to increased risks of mortality and bleeding. These findings challenge the long-standing practice of prophylactic transfusion, which has primarily relied on numerical platelet thresholds.

Thrombocytopenia in dengue is a dynamic and multifactorial process, not merely an isolated quantitative deficiency. A complex hemostatic disturbance arises from the convergence of viral-mediated marrow suppression, immune-driven peripheral destruction, complement activation, platelet consumption, and profound endothelial dysfunction. In this inflammatory environment, transfused platelets are subject to the same consumptive and immunological forces as endogenous platelets, limiting their functional persistence^19^ ^-^ ^21^. More critically, hemorrhagic manifestations in dengue are largely mediated by endothelial injury, glycocalyx disruption, cytokine-driven vascular permeability, and qualitative platelet dysfunction^22^ ^,^ ^23^. Thus, the absence of clinical benefit from platelet transfusion is biologically coherent: correcting the platelet count numerically does not necessarily restore endothelial integrity or correct the broader coagulopathic phenotype.

The observed association between platelet transfusion and increased mortality must be interpreted within the constraints of observational evidence. Confounding by indication is an inherent limitation in transfusion research; patients selected for transfusion often exhibit greater clinical instability, higher perceived bleeding risk, or more severe dengue phenotypes. Such baseline imbalances may inflate adverse outcome rates in the transfused cohort. Nevertheless, the consistency of the mortality signal across studies and the absence of statistical heterogeneity suggest that the association is not driven by isolated datasets. Importantly, platelet transfusion is not physiologically neutral. In the context of dengue, characterized by capillary leak and endothelial permeability, additional intravascular volume may exacerbate hemodynamic instability^24^ ^,^ ^25^. Transfusion-related immunomodulation and inflammatory amplification may further contribute to organ dysfunction. Although causality cannot be definitively established, a plausible biological framework exists through which liberal transfusion strategies could worsen outcomes in susceptible patients^26^.

Perhaps the most significant implication of this analysis is the failure of platelet transfusion to reduce bleeding events, even among patients with profound thrombocytopenia. This finding directly challenges the conventional reliance on platelet count as a surrogate for hemorrhagic risk in dengue. Bleeding in dengue reflects a composite of endothelial disruption, coagulation abnormalities, platelet dysfunction, and inflammatory vascular injury. Platelet count alone poorly captures this multidimensional pathophysiology^19^ ^,^ ^27^. The absence of benefit in low-count subgroups underscores the limitations of threshold-driven transfusion strategies and suggests that numerical targets may represent an oversimplified clinical heuristic rather than a biologically grounded intervention point.

Dengue is endemic in regions where healthcare resources are frequently constrained. Blood products are costly, logistically demanding, and carry inherent risks. In the absence of demonstrable benefit and in the presence of potential harm, routine prophylactic platelet transfusion may constitute low-value care. These findings align with broader movements in transfusion medicine advocating restrictive strategies based on clinical context rather than solely on laboratory thresholds^4^ ^,^ ^17^. From a systems perspective, adopting a more judicious approach to platelet transfusion in dengue may improve resource allocation without compromising patient safety.

The certainty of evidence remains limited by the predominance of non-randomized designs and residual confounding. Variability in transfusion thresholds, heterogeneity in dengue severity classifications, inconsistent bleeding definitions, and differences in supportive care protocols introduce clinical diversity that complicates causal interpretation. Sensitivity analyses indicated that heterogeneity in certain continuous outcomes reflects intrinsic variability across patient populations rather than the disproportionate influence of individual studies. Nonetheless, the scarcity of rigorously designed randomized trials represents a significant gap in the literature. Future investigations should prioritize standardized outcome definitions, stratification by disease phase and severity, and incorporation of biomarkers reflecting endothelial injury and platelet function. Trials should evaluate clinically meaningful endpoints rather than surrogate laboratory targets, thereby aligning research design with pathophysiological insight. 

The cumulative evidence does not support routine prophylactic platelet transfusion in dengue-associated thrombocytopenia based solely on platelet count thresholds. Clinical decision-making should be individualized, emphasizing dynamic assessment of bleeding phenotype, hemodynamic stability, and procedural risk. Transfusion should be reserved for clearly defined high-risk scenarios, such as active major bleeding or invasive interventions where bleeding risk is substantial.

## CONCLUSION

In dengue-associated thrombocytopenia, platelet transfusion does not appear to confer measurable benefit in hematologic recovery or hospitalization and may be associated with adverse outcomes. While residual confounding cannot be fully excluded, the absence of demonstrated efficacy combined with biologically plausible mechanisms of harm argues against liberal prophylactic use. High-quality randomized trials are urgently required to refine transfusion strategies and align clinical practice with contemporary pathophysiological understanding.

## Data Availability

Research data are available in the body of the article in the “Methods” section.
