# Supplementary figures and images for: Platelet Transfusion in Dengue-Associated Thrombocytopenia: A Systematic Review and Meta-Analysis
Source: Rev Soc Bras Med Trop. 2026 Aug 3;59:e0181-2026. doi: 10.1590/0037-8682-0181-2025 (PMC13432798; doi:10.1590/0037-8682-0181-2025)

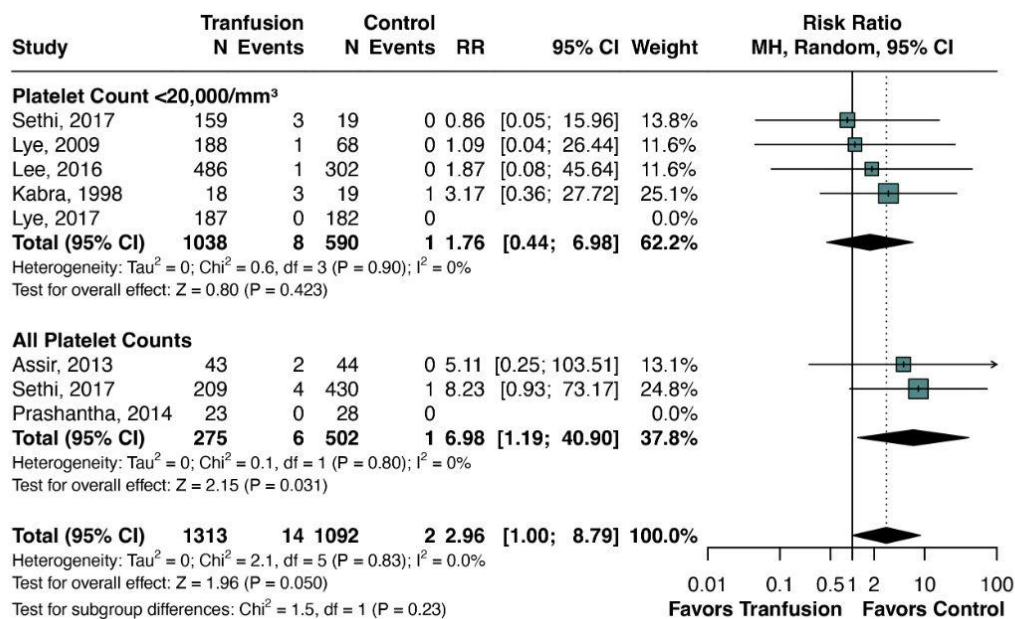

**Supplementary Figure 1.** Forest plot for mortality, stratified by platelet count.

Supplement: Supplementary Figure 1 [file 1678-9849-rsbmt-59-e0181-2026-md1.pdf]

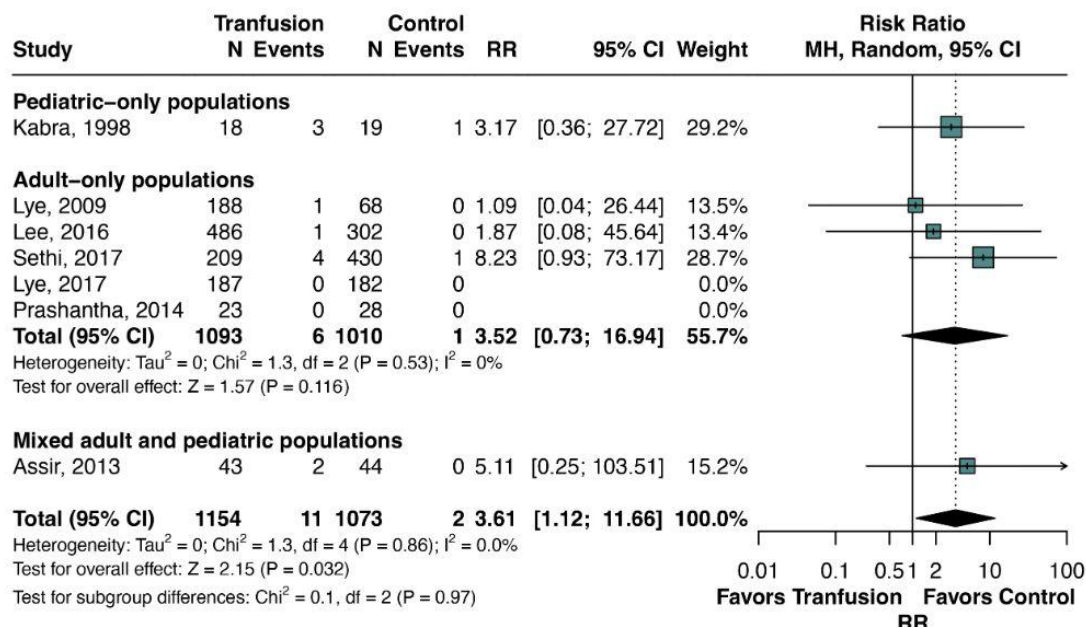

**Supplementary Figure 2.** Forest plot for mortality, stratified by population.

Supplement: Supplementary Figure 2 [file 1678-9849-rsbmt-59-e0181-2026-md2.pdf]

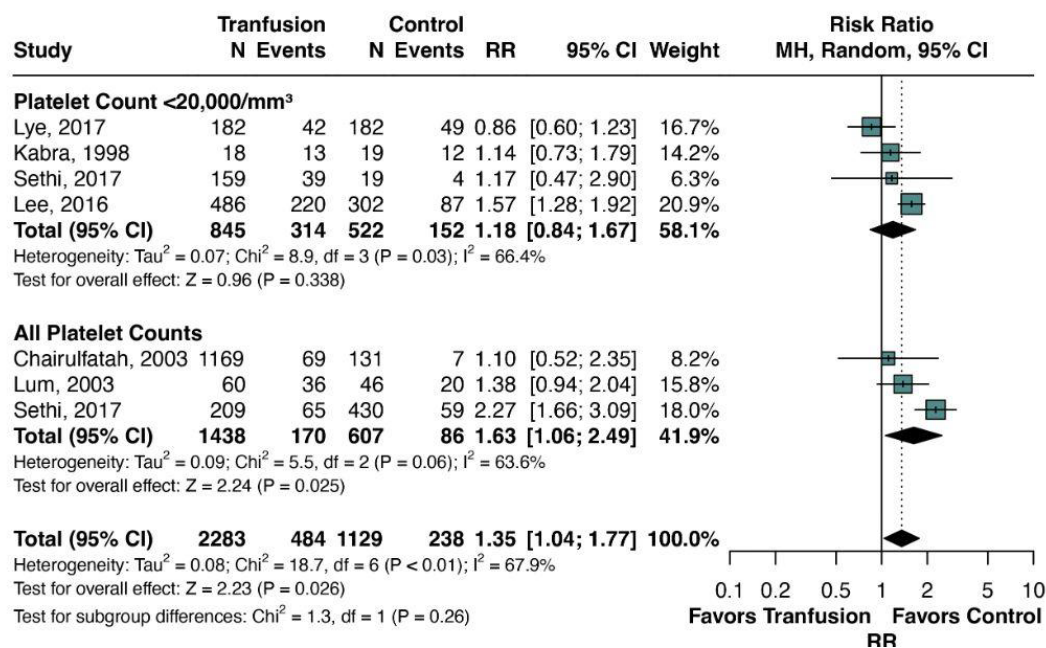

**Supplementary Figure 3.** Forest plot for bleeding events, stratified by platelet count.

Supplement: Supplementary Figure 3 [file 1678-9849-rsbmt-59-e0181-2026-md3.pdf]

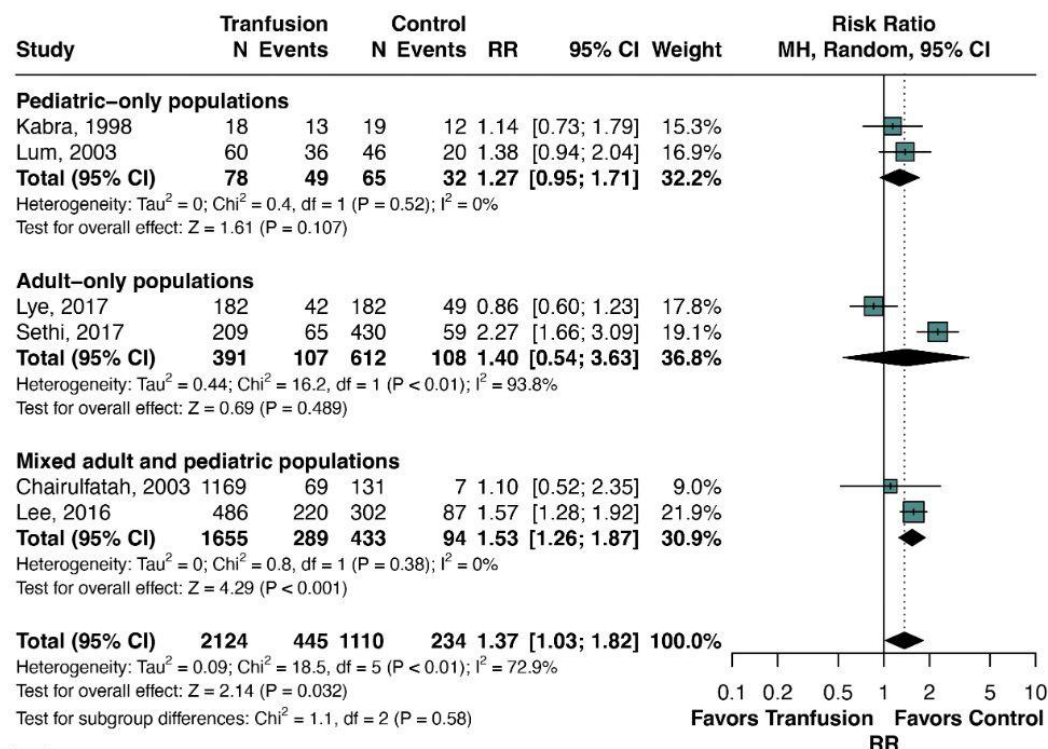

**Supplementary Figure 4.** Forest plot for bleeding events, stratified by population.

Supplement: Supplementary Figure 4 [file 1678-9849-rsbmt-59-e0181-2026-md4.pdf]

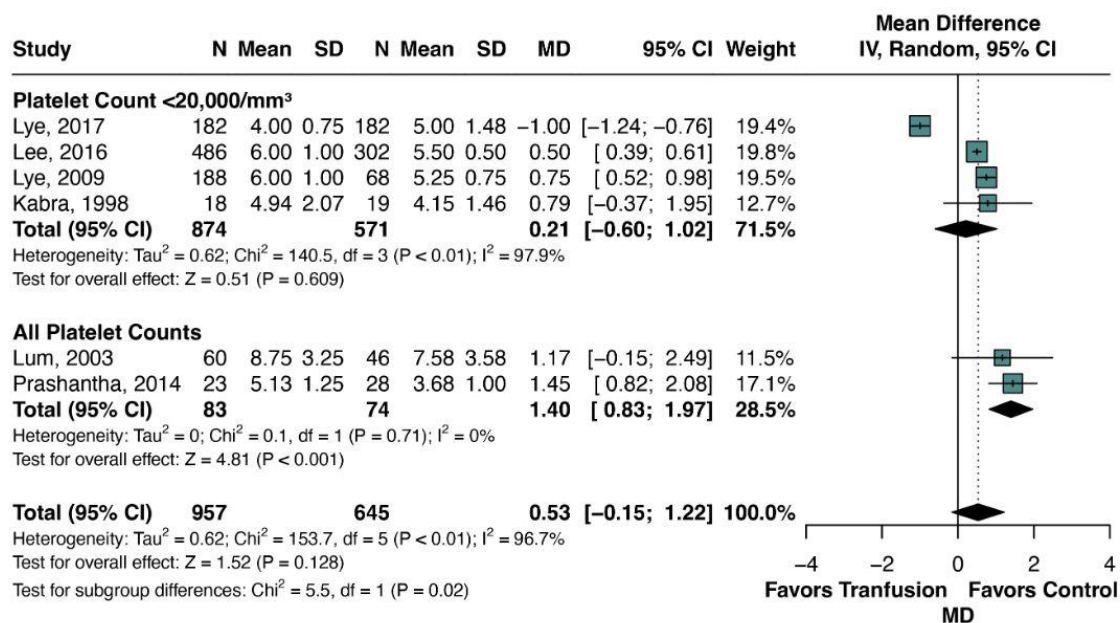

**Supplementary Figure 5.** Forest plot for length of hospital stay, stratified by platelet count.

Supplement: Supplementary Figure 5 [file 1678-9849-rsbmt-59-e0181-2026-md5.pdf]

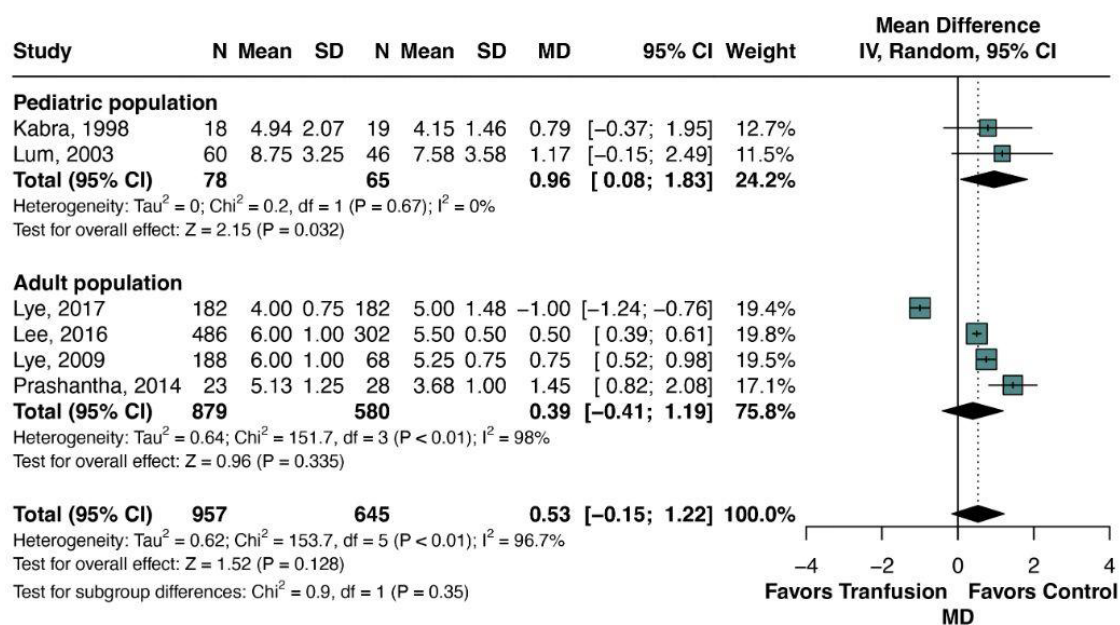

Supplementary Figure 6. Forest plot for length of hospital stay, stratified by population.

Supplement: Supplementary Figure 6 [file 1678-9849-rsbmt-59-e0181-2026-md6.pdf]

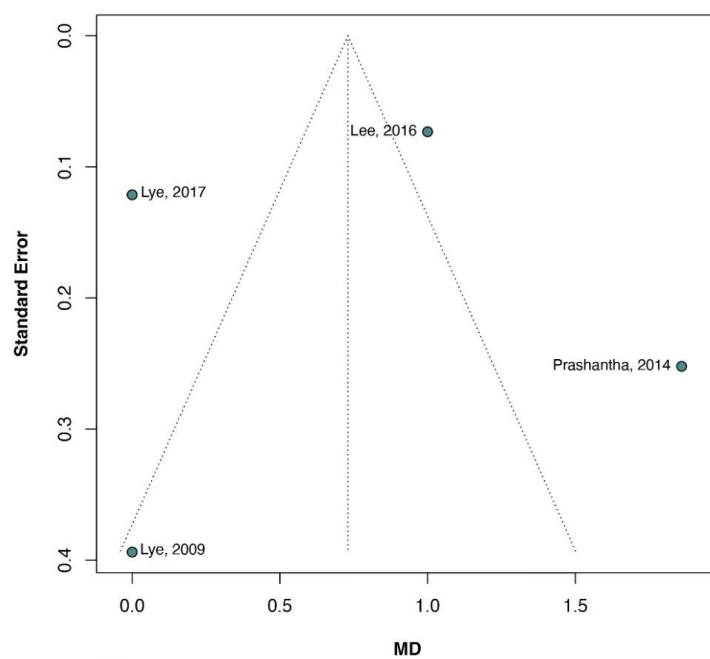

**Supplementary Figure 8.** Baujat plot for time to platelet count  $\geq 50 \times 10^3$  platelets/ $\mu$ L.

Supplement: Supplementary Figure 8 [file 1678-9849-rsbmt-59-e0181-2026-md8.pdf]

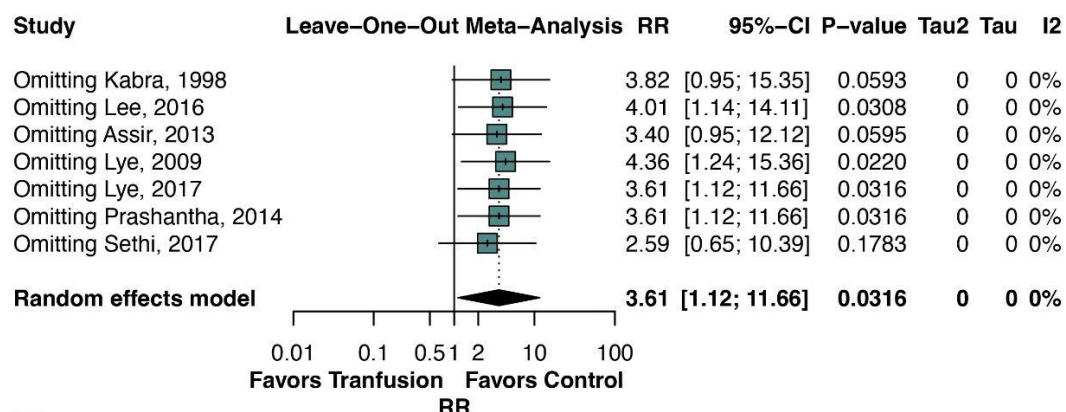

**Supplementary Figure 9.** Leave-one-out sensitivity analysis for mortality.

Supplement: Supplementary Figure 9 [file 1678-9849-rsbmt-59-e0181-2026-md9.pdf]

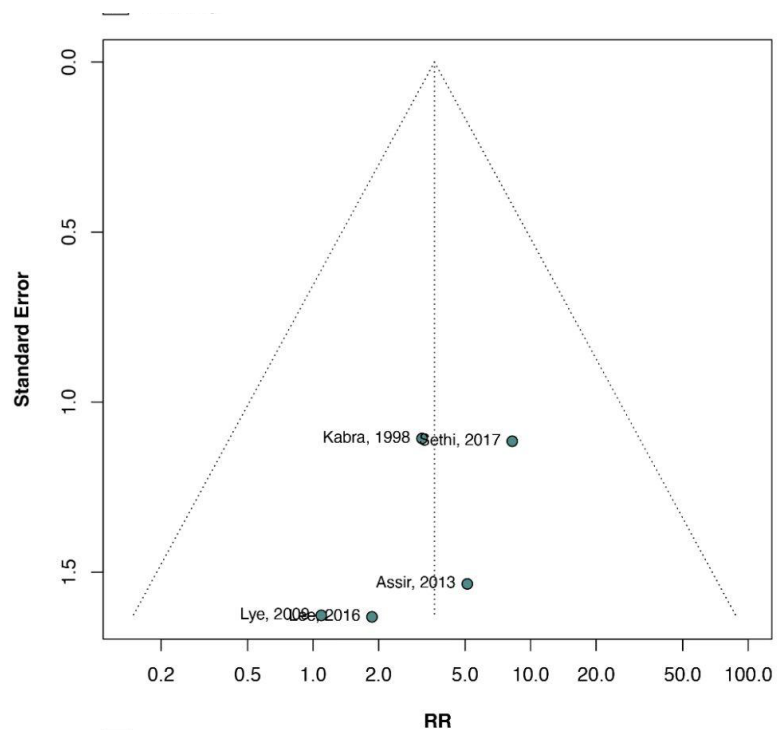

**Supplementary Figure 10.** Baujat plot for mortality.

Supplement: Supplementary Figure 10 [file 1678-9849-rsbmt-59-e0181-2026-md10.pdf]

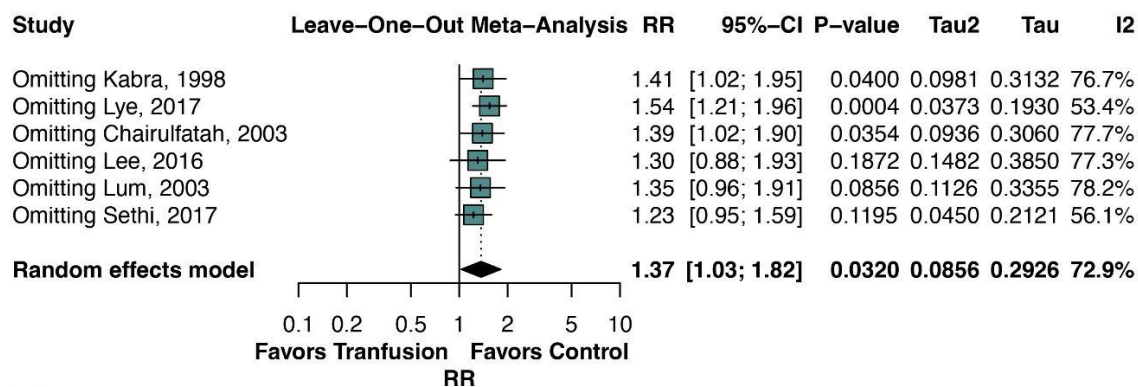

**Supplementary Figure 11.** Leave-one-out sensitivity analysis for bleeding events.

Supplement: Supplementary Figure 11 [file 1678-9849-rsbmt-59-e0181-2026-md11.pdf]

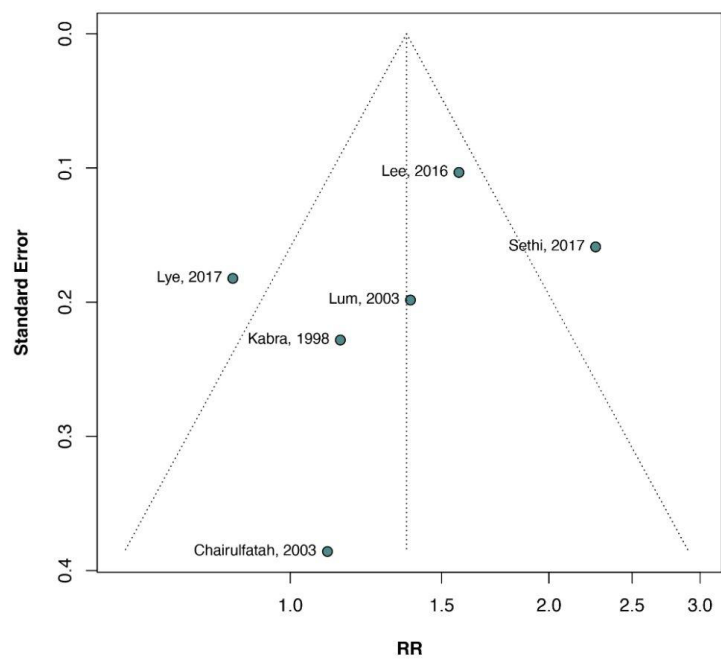

**Supplementary Figure 12.** Baujat plot for bleeding events.

Supplement: Supplementary Figure 12 [file 1678-9849-rsbmt-59-e0181-2026-md12.pdf]

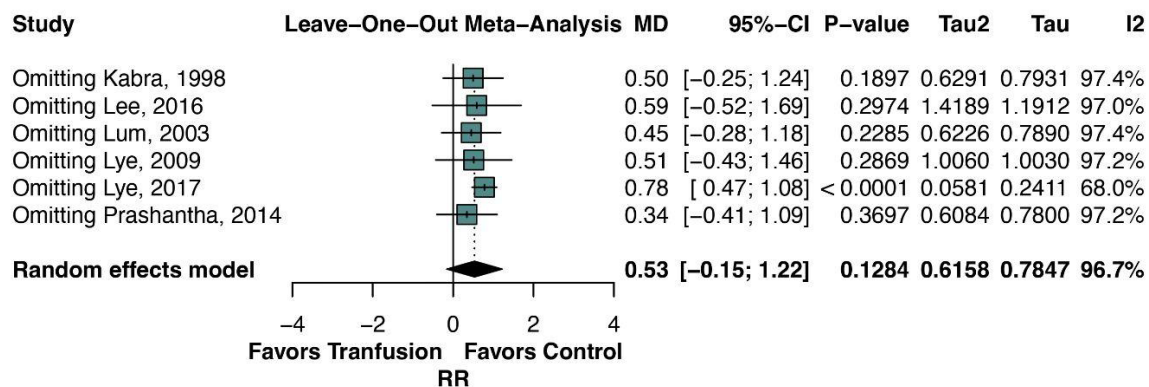

**Supplementary Figure 13.** Leave-one-out sensitivity analysis for length of hospital stay.

Supplement: Supplementary Figure 13 [file 1678-9849-rsbmt-59-e0181-2026-md13.pdf]

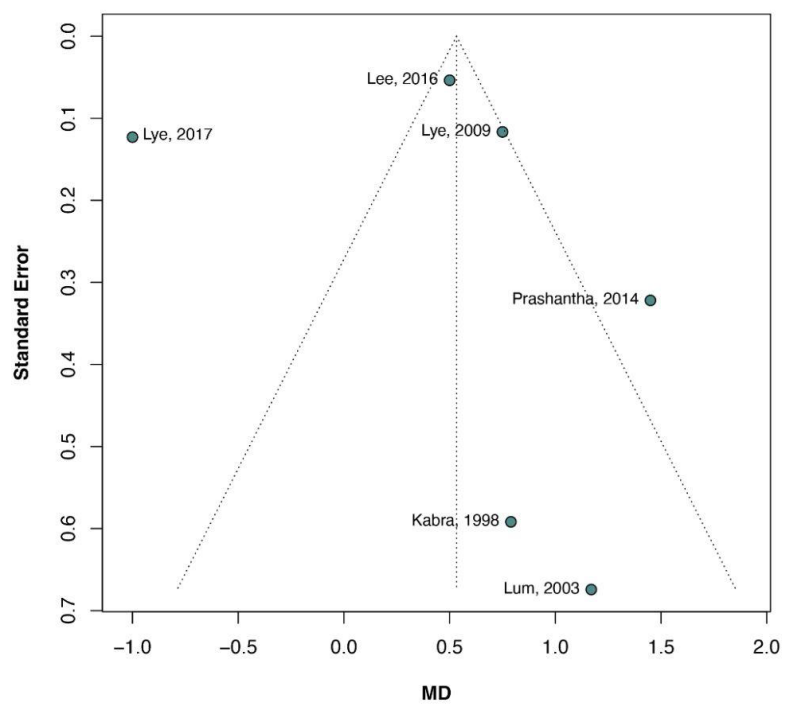

**Supplementary Figure 14.** Baujat plot for length of hospital stay.

Supplement: Supplementary Figure 14 [file 1678-9849-rsbmt-59-e0181-2026-md14.pdf]

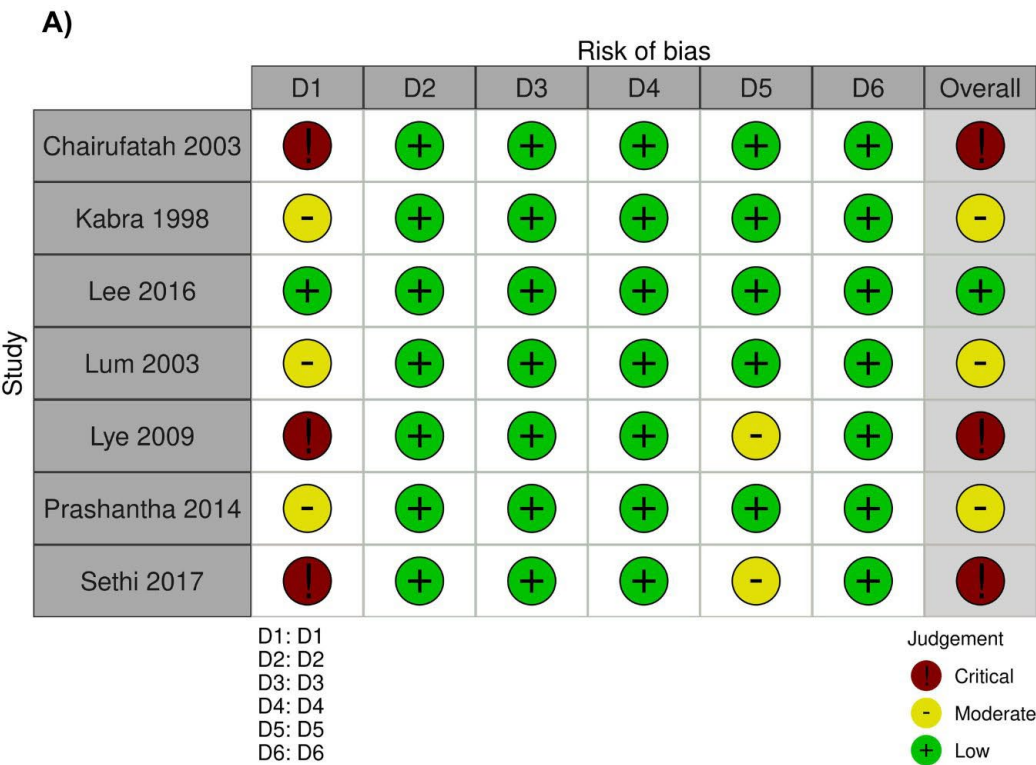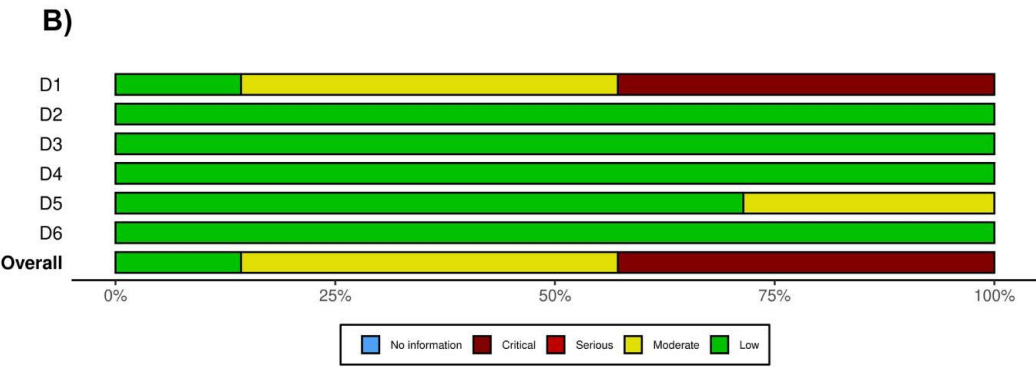

Supplementary Figure 15. ROBINS 1.

Supplement: Supplementary Figure 15 [file 1678-9849-rsbmt-59-e0181-2026-md15.pdf]
