# Supplementary material for: Platelet Transfusion in Dengue-Associated Thrombocytopenia: A Systematic Review and Meta-Analysis
Source: Rev Soc Bras Med Trop. 2026 Aug 3;59:e0181-2026. doi: 10.1590/0037-8682-0181-2025 (PMC13432798; doi:10.1590/0037-8682-0181-2025)
Supplement: Supplementary Figure 16 [file 1678-9849-rsbmt-59-e0181-2026-md16.pdf]

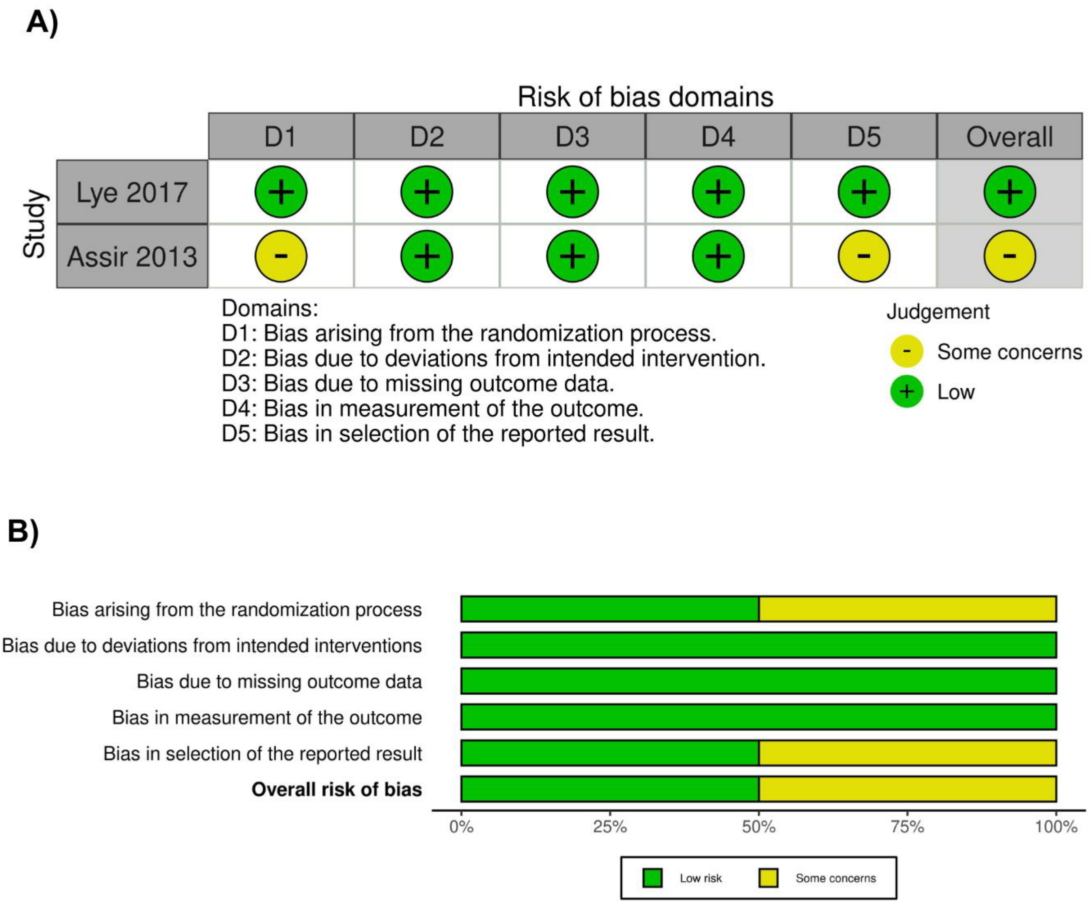

Supplementary Figure 16. Robins 2.
